# Severe Hyponatremia From Lupus-Related Syndrome of Inappropriate Antidiuresis (SIADH): A Diagnostic Challenge

**DOI:** 10.7759/cureus.96577

**Published:** 2025-11-11

**Authors:** Fahad S Alrashidi

**Affiliations:** 1 Internal Medicine and Nephrology, Ad Diriyah Hospital, Riyadh Third Health Cluster, Riyadh, SAU

**Keywords:** 3% hypertonic saline, acute hyponatremia, critical care nephrology, fractional excretion of urate, syndrome of inappropriate secretion of antidiuretic hormone (siadh), systemic lupus erythematosus

## Abstract

Severe hypotonic hyponatremia can complicate systemic lupus erythematosus (SLE) via inflammation-driven non-osmotic vasopressin release, leading to the syndrome of inappropriate antidiuresis (SIADH). We report a 24-year-old woman with active SLE who presented with headache, emesis, and confusion. She was clinically euvolemic, with a serum sodium level of 112 mmol/L, a measured serum osmolality of 254 mOsm/kg, a urine osmolality of 620 mOsm/kg, and a urine sodium level of 64 mmol/L; thyroid and adrenal tests were normal. Neuroimaging was unremarkable. Given severe symptoms, we administered guideline-based 3% hypertonic saline boluses while implementing a proactive desmopressin (DDAVP) clamp to prevent aquaresis-driven overcorrection; targets were an initial rise of 4-6 mmol/L and ≤8-10 mmol/L per 24 hours. Fractional excretion of urate (FEurate) decreased to 5% after partial correction, supporting SIADH over cerebral/renal salt wasting. Sodium was corrected safely with frequent monitoring and a standing re-lowering protocol available if limits were exceeded. This case underscores lupus-related SIADH as an important cause of profound hyponatremia and highlights a practical algorithm - hypertonic saline plus DDAVP clamp, FEurate reassessment, and conservative correction goals - to achieve safety while definitive lupus therapy proceeds.

## Introduction

Hyponatremia is the most common electrolyte disorder in clinical practice, affecting up to 20-30% of hospitalized patients, while severe hypotonic hyponatremia is rare but carries a high risk of seizures, coma, and death [1-3]. Among patients with systemic lupus erythematosus (SLE), hyponatremia has been reported in association with disease activity and neuropsychiatric involvement and may occasionally reflect the syndrome of inappropriate antidiuretic hormone secretion (SIADH) [4]. Inflammation-driven non-osmotic vasopressin release, particularly interleukin-6 (IL-6), provides a plausible mechanism linking active SLE to SIADH [4].

Differentiating SIADH from other causes of hypotonic hyponatremia, such as adrenal insufficiency, drug-induced hyponatremia, and cerebral or renal salt wasting (CSW/RSW), is essential because management strategies differ markedly [1-3,5]. Recent guidelines emphasize classifying hyponatremia by tonicity, severity, and chronicity, and strongly recommend limiting the rate of correction to avoid osmotic demyelination [1-3,6].

We report a young woman with active SLE who presented with profound symptomatic hyponatremia due to lupus-related SIADH. The case highlights a practical diagnostic approach, underscores key discriminators between SIADH and CSW/RSW, and illustrates the safe use of hypertonic saline combined with a proactive desmopressin (DDAVP) clamp.

## Case presentation

This case was managed by the author at Ad-Diriyah Hospital, Riyadh.

A 24-year-old woman with biopsy-proven systemic lupus erythematosus (SLE) presented to the emergency department with 24 hours of progressive headache, nausea, vomiting, and confusion. Her SLE had been diagnosed two years earlier based on American College of Rheumatology (ACR)/European League Against Rheumatism (EULAR) criteria, including malar rash, non-erosive arthritis, positive antinuclear and anti-double-stranded DNA antibodies, hypocomplementemia, and class III lupus nephritis on renal biopsy.

On arrival, she was drowsy but arousable and oriented to person only. Vital signs were as follows: blood pressure 118/72 mmHg, heart rate 92 beats/min, respiratory rate 18 breaths/min, temperature 37.4 °C, and oxygen saturation 98% on room air. She appeared clinically euvolemic, with no orthostatic hypotension, dry mucous membranes, peripheral edema, or ascites. There were no focal neurological deficits or signs of meningeal irritation.

Two weeks before admission, she had a lupus flare characterized by inflammatory polyarthritis and a malar rash with serologic activity (elevated anti-double-stranded DNA (anti-dsDNA) and low complement levels), corresponding to a high Systemic Lupus Erythematosus Disease Activity Index 2000 (SLEDAI-2K) score of 10. This flare was treated with intravenous methylprednisolone pulses followed by high-dose oral prednisolone (1 mg/kg/day). Her chronic medications at the time of presentation included hydroxychloroquine 200 mg twice daily and low-dose oral prednisolone 5 mg/day. Cyclophosphamide induction was planned but had not yet been administered at the time of admission. She was not taking thiazide diuretics, selective serotonin reuptake inhibitors, antiepileptic drugs, carbamazepine, or other agents commonly associated with SIADH. There was no history of recent head trauma, neurosurgery, or central nervous system infection.

Initial laboratory tests showed severe hypotonic hyponatremia with serum sodium 112 mmol/L, potassium 3.7 mmol/L, creatinine 0.6 mg/dL, blood urea nitrogen 9 mg/dL, measured serum osmolality 254 mOsm/kg, urine osmolality 620 mOsm/kg, and urine sodium 64 mmol/L. Serum glucose and lipid profile were normal, excluding pseudohyponatremia and translocational causes. Thyroid-stimulating hormone (1.8 mIU/L; reference range 0.4-4.0 mIU/L) and morning serum cortisol (480 nmol/L) were within the reference range, ruling out hypothyroidism and adrenal insufficiency. Serum uric acid was low at 2.1 mg/dL. Brain computed tomography and magnetic resonance imaging showed no acute intracranial pathology.

Urine output and hemodynamics were closely monitored during the first 12 hours and remained stable, supporting clinical euvolemia rather than hypovolemia. The fractional excretion of urate (FEurate) was calculated as $\frac{U_{\text{urate}} \times P_{\text{creatinine}}}{P_{\text{urate}} \times U_{\text{creatinine}}} \times 100$ and, after partial correction of serum sodium to 116-118 mmol/L, declined to 5%, which is within the normal range (4-11%) and therefore strongly favored SIADH over cerebral or renal salt wasting, in which FEurate typically remains >11% despite correction.

Taken together - hypotonic hyponatremia, inappropriately concentrated urine, elevated urine sodium in an apparently euvolemic and hemodynamically stable patient, normal thyroid and adrenal function, absence of offending drugs, and normalization of FEurate after partial correction - the findings were consistent with lupus-related SIADH (Table 1).

**Table 1:** Key diagnostic data and discriminators between SIADH and CSW/RSW FEurate, fractional excretion of urate; Uosm, urine osmolality; UNa, urine sodium; CSW, cerebral salt wasting; RSW, renal salt wasting; TSH, thyroid-stimulating hormone; SIADH: syndrome of inappropriate antidiuresis

Parameter	Patient	SIADH (expected)	CSW (expected)	Comment
Serum Na (mmol/L)	109	↓	↓	Severe hyponatremia at presentation
Serum osmolality (mOsm/kg)	232	↓ (<275)	↓ (<275)	Hypotonic
Urine osmolality (mOsm/kg)	620	↑ (>100)	↑ (>100)	Inappropriately concentrated
Urine Na (mmol/L)	76	↑ (>30)	↑ (>30)	Both can be high
Clinical volume	Euvolemic	Euvolemia	Hypovolemia	No signs of depletion
Serum uric acid (mg/dL)	2.1	↓	↓	Low in both initially
FEurate (%) after correction	5 %	Normalizes (4–11 %)	Stays > 11 %	Helps distinguish
TSH / Cortisol	Normal	Normal	Normal	Endocrine causes excluded
Clinical volume	Euvolemic	Euvolemia	Hypovolemia, often with unstable hemodynamics	No signs of volume depletion or instability in our patient

Differential diagnosis

Key mimics included cerebral/renal salt wasting (CSW/RSW), adrenal insufficiency, and thiazide‑associated hyponatremia. Clinical euvolemia and urine indices (Uosm >100 mOsm/kg, UNa >30 mmol/L) supported SIADH. After partial correction to serum sodium 116-118 mmol/L, FEurate declined to 5%, favoring SIADH; persistent FEurate >11% would suggest CSW/RSW. This distinction is clinically crucial because fluid restriction, appropriate for SIADH, may worsen hypovolemia in CSW/RSW. If cyclophosphamide is used, early sodium checks within 4-12 hours are advised due to the risk of drug‑induced SIADH.

Management

Given severe symptoms, we administered 3% hypertonic saline boluses according to guideline recommendations - European: 150 mL up to 3 times over 20 minutes; US: 100 mL up to 3 times over 10 minutes - aiming for an initial 4-6 mmol/L rise while limiting total correction to ≤10 mmol/L at 24 hours. A proactive desmopressin clamp (1-2 µg IV every 6-8 h with titrated 3% saline) was implemented to avoid overcorrection, supported by ICU and nephrology literature [7]. We targeted ≤8 mmol/L per 24 hours, given potential high‑risk features for osmotic demyelination. Electrolytes were monitored every 2-4 hours with a standing re-lowering protocol, should limits be exceeded: 2 µg IV desmopressin plus D5W infusion (~10 mL/kg), with repeat sodium checks every 2 hours until within target. Chronic control included fluid restriction (~500 mL/day below 24‑hour urine output) and oral urea; vaptans were deferred due to cost, safety profile, and the risk of rapid aquaresis in this context. Lupus therapy was intensified with corticosteroids and immunosuppression.

Given her high SLEDAI-2K score and serologic activity, lupus-directed immunosuppression had recently been intensified with three daily pulses of intravenous methylprednisolone (500 mg/day) followed by high-dose oral prednisolone (1 mg/kg/day), in addition to maintenance hydroxychloroquine 200 mg twice daily. At the time of admission with severe hyponatremia, no other immunosuppressive agents (such as cyclophosphamide, mycophenolate mofetil, or biologic therapy) had yet been started.

During the acute SIADH episode, management focused on controlled correction of hyponatremia rather than further escalation of immunosuppression. Cyclophosphamide induction was deliberately deferred until after serum sodium had been safely stabilized. In contrast, the recent lupus flare two weeks earlier had been managed primarily with corticosteroid escalation alone, without the constraints imposed by severe electrolyte disturbance. This distinction may help clinicians balance the urgency of treating active SLE against the risk of over-treating during a high-risk hyponatremic state.

## Discussion

This case highlights key diagnostic and management principles for severe hypotonic hyponatremia in SLE, a challenging and under-recognized complication. Inflammation-driven vasopressin release (e.g., IL-6) and neuro-SLE provide a biologic basis for SIADH. Safety hinges on respecting correction limits; both European and U.S. guidance emphasize hypertonic saline boluses with frequent reassessment, and endocrine emergency recommendations reiterate strict 24-hour limits for a sodium rise [1,2,4,5].

**Figure 1:**
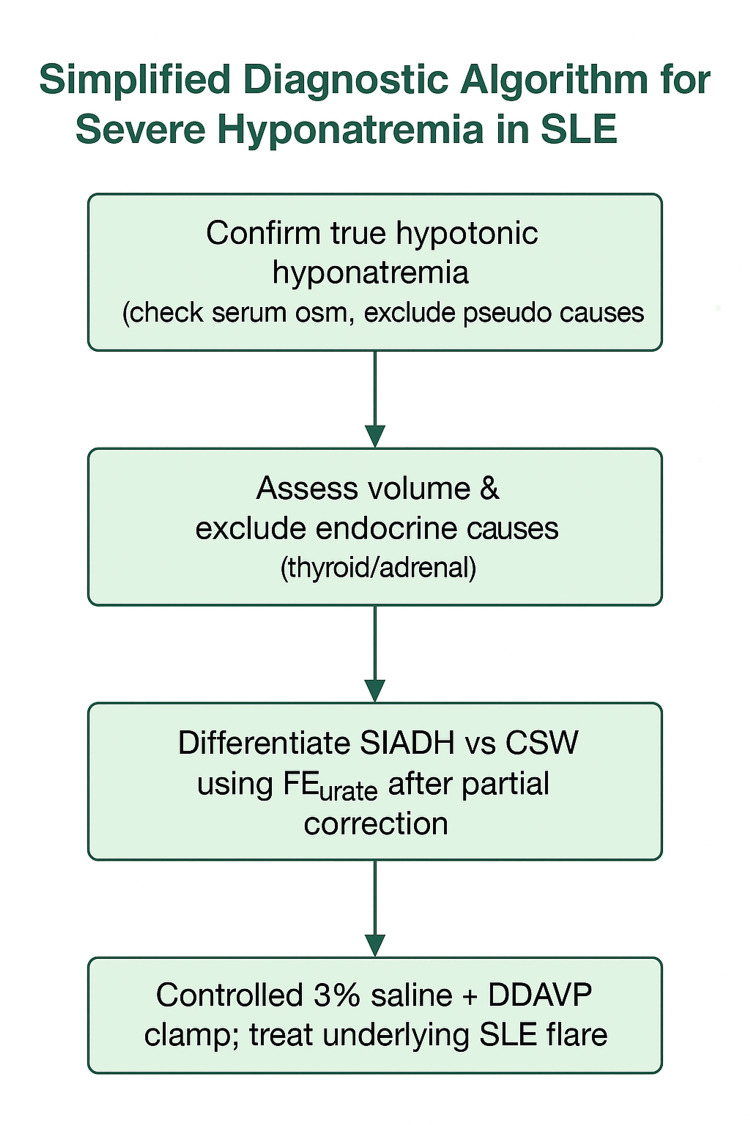
Diagnostic approach to severe hypotonic hyponatremia in SLE Confirm measured hypotonicity and exclude pseudohyponatremia/translocational causes (glucose/lipids) before assigning SIADH; consider a DDAVP clamp with titrated 3% saline; reassess sodium every 2-4 h; re‑lower proactively if limits are exceeded. SLE: systemic lupus erythematosus; SIADH: syndrome of inappropriate antidiuretic hormone secretion; CSW: cerebral salt wasting; FEurate: fractional excretion of urate; DDAVP: desmopressin

Established osmotic demyelination risk factors, including very low baseline sodium, hypokalemia, malnutrition, alcoholism, and liver disease, guided our conservative correction targets [8-11]. The stepwise diagnostic and therapeutic framework for lupus-related hyponatremia is summarized in Figure 1, which provides a concise bedside guide for clinicians managing SLE-associated SIADH.

## Conclusions

In patients with systemic lupus erythematosus who present with severe hypotonic hyponatremia, the syndrome of inappropriate antidiuretic hormone secretion should be strongly considered only after careful exclusion of endocrine causes (adrenal insufficiency and hypothyroidism) and drug-induced hyponatremia (e.g., thiazide diuretics, selective serotonin reuptake inhibitors, and antiepileptic agents, including carbamazepine and cyclophosphamide). Routine early calculation of the fractional excretion of urate and the use of a predefined re-lowering protocol are practical safeguards to reduce the risk of osmotic demyelination. A structured diagnostic approach with cautious sodium correction, often facilitated by a desmopressin clamp, optimizes safety while definitive therapy for active lupus is instituted.
